# Therapy of Organophosphate Poisoning via Intranasal Administration of 2-PAM-Loaded Chitosomes

**DOI:** 10.3390/pharmaceutics14122846

**Published:** 2022-12-19

**Authors:** Elmira A. Vasilieva, Darya A. Kuznetsova, Farida G. Valeeva, Denis M. Kuznetsov, Andrey V. Zakharov, Syumbelya K. Amerhanova, Alexandra D. Voloshina, Irina V. Zueva, Konstantin A. Petrov, Lucia Ya. Zakharova

**Affiliations:** Arbuzov Institute of Organic and Physical Chemistry, FRC Kazan Scientific Center, Russian Academy of Sciences, Arbuzov Str. 8, 420088 Kazan, Russia

**Keywords:** chitosomes, arginine chitosan, acetylcholinesterase reactivation, intranasal delivery, pralidoxime chloride, organophosphorus compounds

## Abstract

Chitosan-decorated liposomes were proposed for the first time for the intranasal delivery of acetylcholinesterase (AChE) reactivator pralidoxime chloride (2-PAM) to the brain as a therapy for organophosphorus compounds (OPs) poisoning. Firstly, the chitosome composition based on phospholipids, cholesterol, chitosans (Cs) of different molecular weights, and its arginine derivative was developed and optimized. The use of the polymer modification led to an increase in the encapsulation efficiency toward rhodamine B (RhB; ~85%) and 2-PAM (~60%) by 20% compared to conventional liposomes. The formation of monodispersed and stable nanosized particles with a hydrodynamic diameter of up to 130 nm was shown using dynamic light scattering. The addition of the polymers recharged the liposome surface (from −15 mV to +20 mV), which demonstrates the successful deposition of Cs on the vesicles. In vitro spectrophotometric analysis showed a slow release of substrates (RhB and 2-PAM) from the nanocontainers, while the concentration and Cs type did not significantly affect the chitosome permeability. Flow cytometry and fluorescence microscopy qualitatively and quantitatively demonstrated the penetration of the developed chitosomes into normal Chang liver and M-HeLa cervical cancer cells. At the final stage, the ability of the formulated 2-PAM to reactivate brain AChE was assessed in a model of paraoxon-induced poisoning in an in vivo test. Intranasal administration of 2-PAM-containing chitosomes allows it to reach the degree of enzyme reactivation up to 35 ± 4%.

## 1. Introduction

A large number of neurotropic substances are known to have a toxic effect on the central nervous system (CNS) due to a violation of the catalytic function of acetylcholinesterase (AChE) [1,2]. The AChE rapidly hydrolyzes an excess of the neurotransmitter acetylcholine preventing its accumulation in cholinergic synapses, thus providing a balance between the inhibition and excitation processes. Anticholinesterase compounds are widely used in medicine and agriculture [3,4,5]. It is known that organophosphorus compounds (OPs) are their toxic representatives, which can be used as chemical weapon components. According to the World Health Organization, there are approximately 3 million accidental organophosphate poisonings/self-poisonings and over 200,000 deaths worldwide every year [6]. OP poisoning leads to a cholinergic crisis that is accompanied by bronchospasm, increased salivation, muscle fasciculations, respiratory depression, loss of consciousness, and epileptic seizures [7,8]. Currently, the treatment of patients with OP poisoning includes the use of anticholinergic medications (atropine, benztropine, etc.), anticonvulsants (benzodiazepines), and the protection of AChE from irreversible inhibition and its reactivation [9]. AChE reactivators are excellent antidotes since they are able to accelerate the recovery process of catalytic activity of the enzyme inhibited by OPs [10]. The most common antidotes are chemical compounds containing an oxime group. The nucleophilic attack of the oxime on the phosphylated enzyme leads to the breaking of bonds between the phosphorus atom of the OP and the oxygen of serine in the blocked active center of AChE. The result of such an interaction is the formation of the free enzyme and phosphylated oxime. Pyridine-2-aldoxime (2-PAM) is the only reactivator of AChE approved by the FDA (US Food and Drug Administration), which is most commonly used to treat OP poisoning compared with other oximes (obidoxime, HI-6) [11,12]. Nevertheless, 2-PAM shows a low degree of phosphylated AChE reactivation in the CNS due to limited ability to cross the blood–brain barrier (BBB). Such a disadvantage is associated with the hydrophilicity of the oxime and its permanent positive charge [10]. There are several main directions for improving oxime therapy: an active search for new, more effective reactivators [13,14,15,16] and the development of oxime formulations (including nanocarriers) that ensure their passage through biological barriers, i.e., BBB [17,18]. The second strategy has been widely studied and described in a previous review [19].

Intranasal drug administration is one of the non-invasive methods to treat brain diseases due to the rapid delivery of drugs to the target [20,21]. The nasal mucosa (in the olfactory region) can provide a direct route for drugs to the brain, bypassing the BBB [22]. Furthermore, the nasal cavity is highly vascularized (in the respiratory region), so active substances can immediately enter the systemic circulation. Drugs can reach the CNS by paracellular or transcellular transport via the olfactory mucosa, as well as through the trigeminal pathway [22]. The intranasal administration of drugs avoids their hepatic metabolism, which improves treatment effectiveness [23]. However, mucociliary clearance, poor penetration of hydrophilic substances, restricted administration volumes, and enzymatic degradation are limiting factors of the intranasal route of drug administration. Liposomes are great drug delivery systems due to their targeting ability, the encapsulation of both lipid-soluble and water-soluble drugs, biocompatibility and low immunogenicity [24,25,26]. Unfortunately, conventional liposomes have low stability and a tendency to aggregate and fuse, which leads to drug leakage during storage. Positively charged compounds can increase retention of the liposomes in the nasal cavity due to electrostatic interactions between nanoparticles and mucosal components [21]. In the case of liposomes, a positive charge can be achieved by the introduction of cationic surfactants [17,27,28,29], lipids, or polymers [30]. There are many publications devoted to covering liposomes with different types of chitosan (Cs), which can be used to modify their functionality and are aptly described in recent reviews [31,32]. A polymer coating can improve the structural properties and physicochemical stability of liposomes [31]. In addition, polymers can act as a barrier to control the release rate of encapsulated drugs due to the formation of a hydrophilic steric layer around the liposomes [33,34]. These kinds of nanocontainers are exemplified in this paper by chitosomes formed due to the electrostatic interaction of liposomes and Cs [32,33,35]. Cs and its derivatives are successfully used as a liposome shell for drug delivery through the mucous membranes of the nose [36], eyes [37], mouth [38], and digestive system [39]. Cs can interact with negatively charged fragments, mainly with sialic acid in the mucous layers, due to its positively charged amino groups [40]. The mucoadhesive property of Cs is associated with the penetration of the polymer into glycoprotein mucin chains and the formation of tangles with mucus. Such an interaction increases both the retention of the drug in the nasal cavity and the penetration of active substances through the mucosa. The interest in Cs formulations in the context of intranasal drug delivery is due to its mucoadhesive properties and the ability to temporarily open tight junctions between epithelial cells in the olfactory and respiratory regions [41,42]. It was shown that Cs causes disruption of tight junctions on monolayers of Caco-2 (human colorectal adenocarcinoma) cells due to translocation into the cytosol of the membrane (occludin, claudin) and cytosolic proteins (ZO-1) [41,43]. In another study, the authors, using a number of microscopy methods, showed that Cs has a temporary effect on the permeability of tight junctions without affecting cell viability [44]. Nasal histology studies have shown that the use of Cs solutions for 7 days does not cause harmful effects on the nasal mucosa [45]. In general, the properties of Cs are widely studied and presented in many works. In addition, this polysaccharide is approved by the FDA and EMA (European Medicines Agency) [46]. Cs is also used as an enhancer of transcellular and paracellular transport of drugs both in vitro and in vivo [43].

It should be noted that Cs can be functionalized by introducing ligands and substituents due to the presence of amino and hydroxy-groups in the polymer. This makes it possible to improve the functional properties of the polymer, which, in turn, expands the practical application of chitosomes. However, Cs loses its charge and becomes insoluble at physiological pH. To overcome this limitation, Cs can be modified with arginine to acquire a positive charge at a neutral pH [47,48]. In addition, it was shown that the content of arginine in peptides facilitates and enhances their uptake by HeLa WT eukaryotic cells [49]. Such an effect is caused by the presence of a guanidine group in the arginine structure and is not only due to the positive charge. The guanidine group forms bidentate hydrogen bonds with negatively charged phosphate, sulfate, and carboxylate groups on the cell surface [50].

In most cases, the coating of the liposome surface with Cs occurs on preformed lipid vesicles, while only the outer layer of the membrane is available for polymer deposition. However, there are methods in which both sides of the phospholipid bilayer can be coated with Cs [51]. Recent reviews describe the benefits of combining liposomes with a Cs coating to overcome the disadvantages of conventional liposomal formulations [31,32,52].

**Figure 1:**
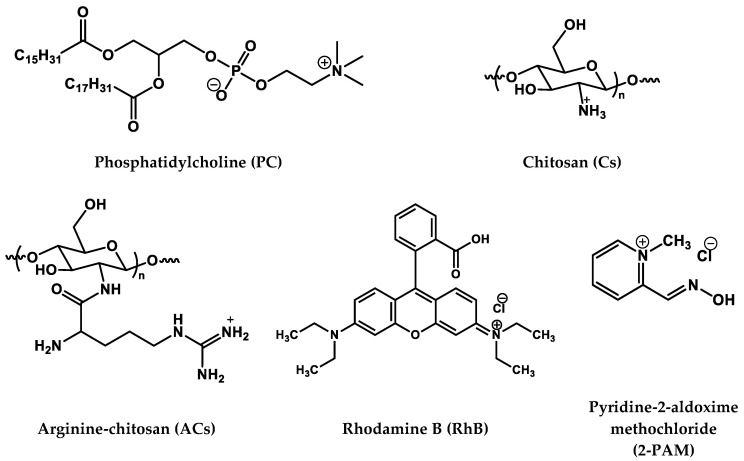
Chemical structure of lipid, polymers, and substrates.

In view of the above, Cs coating is a promising liposome modification strategy for the retention of nanocontainers in the nasal cavity and the transport of hydrophilic molecules to the brain [53]. Therefore, the purpose of this work is to decorate the surface of liposomes with Cs for delivery of the AChE reactivator 2-PAM using the “from nose to brain” strategy. The structures of the compounds used are shown in Figure 1. This work is the first example of using Cs-decorated liposomes (chitosomes) for the intranasal delivery of AChE reactivator 2-PAM as an oxime therapy for OP poisoning. Moreover, the arginine derivative of Cs was used as a shell of the liposome for the first time. Within the framework of this study, a variety of physicochemical assays were caried out followed by in vitro and in vivo tests. To begin with, the composition of formulations was optimized to achieve the stability of nanoparticles, for which the physicochemical characteristics, including shape, hydrodynamic diameter, and zeta potential were monitored by dynamic/electrophoretic light scattering over a long-term period and visualized with transmission electron microscopy. The diffusion rate of 2-PAM through the chitosome membrane was estimated in vitro by spectrophotometry by varying the Cs concentration and Cs modification. The cellular internalization of chitosomes was studied by fluorescence microscopy and flow cytometry using normal and cancer cell lines as examples. The hemolytic activity and cytotoxicity of the developed nanosized vesicles were evaluated. For the first time, in vivo experiments were carried out to determine the degree of AChE reactivation during intranasal administration of 2-PAM encapsulated in chitosomes. The results obtained were analyzed and compared with previously published data on AChE reactivation in vivo through intranasal administration of 2-PAM-nanoparticles [54].

## 2. Materials and Methods

### 2.1. Materials

Soybean L-α-phosphatidylcholine (95%, MW = 770 g/mol) was purchased from Avanti Polar Lipids, Inc. (Alabaster, AL, USA). Low-molecular-weight Cs (50–190 kDa, 75–85% deacetylated), rhodamine B (RhB, ≥95%), pyridine-2-aldoxime methochloride (2-PAM, ≥97%), and Boc-L-arginine were purchased from Sigma–Aldrich (St. Louis, MO, USA). All systems were prepared using purified water from the Milli-Q Millipore system (Millipore S.A.S. 67120 Molsheim, France) with a resistivity of 18 MΩ cm.

### 2.2. Synthesis and Characterization of Arginine-Chitosan

In the first stage, acid degradation of Cs with a molecular weight of 50–190 kDa was performed as follows: 1 g of Cs was dissolved in HCl (C = 4 M) and left for 48 h in a water bath with constant stirring (500 rpm). After that, the final solution was dialyzed (MWCO = 12.5 kDa) in distilled water for 48 h and then lyophilized (Alpha 2-4 LD plus, Martin Christ Gefriertrocknungsanlagen GmbH, Osterode am Harz, Germany). The weight average (Mw), number average (Mn), and relative molecular weight, as well as the polydispersity index (PdI) of Cs, were determined by high-performance gel permeation chromatography (HP-SEC) on a Shodex OHpak SB-806M HQ column (8.0 mm × 300 mm, Showa Denko America, Inc., New York, NY, USA). The column temperature and flow rate were maintained at 50 °C and 0.3 mL/min, respectively. Deionized water was used as the eluent. The measurements were performed using a refractive index detector (Agilent Technologies, Santa Clara, CA, USA), the results of which were analyzed using the Agilent GPC/SEC software Version A:02:01.

Next, the low-molecular-weight Cs obtained was modified with L-arginine according to Lv et al. [55]. Activation of the carboxyl group of Boc-L-arginine was carried out as follows: 0.5 g of Boc-L-arginine dissolved in bidistilled water, N-hydroxysuccinimide (NHS), and 1-ethyl-3-(3-dimethylaminopropyl) carbodiimide (EDC) were mixed at a molar ratio of 1:1:3, respectively. The pH of the solution was then adjusted to pH 6 using acetic acid (1%) and the NaOH solution (1%). The reaction was carried out for 2 h. Next, 0.73 g of Cs (MW = 50 kDa) was dissolved in 1% acetic acid (50 mL) and the pH was adjusted to 6 using 1% NaOH. An aqueous solution of Boc-L-arginine with an activated carboxyl group was then added to the Cs solution. The reaction proceeded for 48 h at room temperature with continuous stirring (250 rpm). At the end of the reaction, the Boc groups were removed using 1% CF_3_COOH. The resulting reaction mixture was subjected to dialysis for 48 h in dialysis bags with a molecular weight cutoff of 3.5 kDa and then lyophilized (Martin Christ alpha 2-4 LD plus, Martin Christ Gefriertrocknungsanlagen GmbH, Osterode am Harz, Germany).

The lyophilized form of the modified Cs, as well as the original one, was analyzed using Fourier transform infrared (Tensor 27 FTIR spectrometer, Bruker, Ettlingen, Germany) in the range of 400–4000 cm^−1^. For this, KBr pellets were used, and 20 scans per sample were performed (resolution is 4 cm^−1^). The IR spectra of the modified arginine-chitosan were compared to the original Cs to confirm the chemical bond (Figure S1). The degree of substitution (DS) of the ACs was determined by elemental analysis of C, H, and N (CHNS analyzer EuroEA3028-HT-OM (Eurovector SpA, Redavalle PV, Italy)) according to Equation (1) [47]:

(1) $$\mathrm{DS}\;\left(\%\right)=\frac{{\left(C/N\right)}_{\mathrm{ACs}}-{\left(C/N\right)}_{\mathrm{Cs}}}{n}$$

where ${\left(C/N\right)}_{\mathrm{ACs}}$ is the percentage relation of C and N for the ACs, ${\left(C/N\right)}_{\mathrm{Cs}}$ is the percentage relation for Cs, and n is the number of arginine carbons. The resulting elemental analysis percentages and DS are shown in Table 1.

### 2.3. Preparation of Chitosomes

Empty and RhB/2-PAM-loaded liposomes were prepared using the thin lipid film hydration method as previously reported [56]. The concentration of RhB and 2-PAM for loading into chitosomes was 0.5 mg/mL and 10 mg/mL, respectively. Cs (MW = 50–190 kDa) was dissolved in acetate buffer (pH 4.4) for chitosome preparation (F1). The Cs solution at different concentrations (0.1–1%) was added dropwise to an empty or substrate-loaded liposome dispersion (at a ratio of 1:1) at room temperature for 5 h under constant stirring (250 rpm). The suspension was incubated overnight at 4 °C to stabilize the dispersion. Chitosomes with Cs (50 kDa) and ACs (50 kDa) were prepared according to the same procedure, except for replacing the acetate buffer with water, and were labeled F2 and F3, respectively.

### 2.4. Vesicle Size, Zeta Potential and PdI

The hydrodynamic diameter (D_h_), zeta potential (ζ), and PdI of polymer-coated liposomes were determined by dynamic and electrophoretic light scattering (DLS/ELS) using a Zetasizer Nano (Malvern Instruments Ltd., Worcestershire, UK). All measurements were carried out in triplicate at 25 °C in a folded capillary zeta cell. All chitosome samples were diluted to 1 mM before measurements. The D_h_ and ζ were accomplished according to the Stokes–Einstein [57] and Helmholtz Smoluchowski [56,58] equations, respectively. The PdI was calculated with a second-order cumulant analysis of the correlation functions.

### 2.5. Encapsulation Efficiency

Encapsulation efficiency (EE) was determined as follows: 0.3 mL of the chitosome dispersion was placed in an Amicon ultracentrifugal filter (molecular weight cutoff is 100 kDa, Amicon Ultra-0.5, Merck Millipore, Darmstadt, Germany) and centrifuged at 10,000 rpm for 10 min to separate the free (unloaded) RhB and 2-PAM. The concentration of non-encapsulated RhB and 2-PAM was calculated from the optical density of the substrates at the maximum absorption (554 nm and 294 nm, respectively) using Specord 250 Plus (Analytik Jena AG, Jena, Germany). EE was calculated according to Equation (2):

(2) $$\mathrm{EE}=\frac{\mathrm{Total}\;\mathrm{amount}\;\mathrm{of}\;\mathrm{drug}-\mathrm{free}\;\mathrm{drug}}{\mathrm{Total}\;\mathrm{amount}\;\mathrm{of}\;\mathrm{drug}}\times 100\%$$

### 2.6. In Vitro Release of Rhodamine B/2-PAM from Chitosomes

The in vitro RhB or 2-PAM release from chitosomes was determined using dialysis bags (Zellu Trans Dialysis Tube T2 MWCO 3500 Da, Scienova GmbH, Jena, Germany). One milliliter of RhB- or 2-PAM-loaded chitosomes was added to the dialysis bag, which was placed in a phosphate buffer (pH 7.4) at 37 °C under constant stirring (250 rpm). Samples of 2.7 mL were taken at certain time intervals until the RhB or 2-PAM release stopped. Absorption spectra of RhB and 2-PAM were recorded (Specord 250 Plus, Analytik Jena AG, Jena, Germany) at 554 nm and 294 nm, respectively. The drug release profile was fitted to zero-order, Korsmeyer–Peppas, and Higuchi models to determine the release kinetic from chitosomes.

### 2.7. Hemolytic Activity

The hemolytic activity of chitosomes was assessed via the ability of particles to destroy erythrocytes (group IV). The hemolysis degree was determined by comparing the optical density of hemoglobin at 100% hemolysis with the optical density of hemoglobin after applying the chitosome dispersion. A detailed description of the methodology can be found in [17].

### 2.8. Cytotoxicity Assay and Cellular Uptake Studies

The cytotoxic effect of chitosomes was studied using the colorimetric method of cell proliferation—the MTT test. NADP-H-dependent cellular oxidoreductase enzymes can reflect the number of viable cells under certain conditions. These enzymes are able to reduce the tetrazolium dye (MTT)-3-(4,5-dimethylthiazol-2-yl)-2,5-diphenyl-tetrazolium bromide to insoluble blue-violet formazan, which crystallizes inside the cell. The amount of formazan formed is proportional to the number of cells with an active metabolism. Cells were seeded on a 96-well Nunc plate at a concentration of 5 × 103 cells per well in a volume of 100 μL of medium and cultured in a CO_2_ incubator at 37 °C until a monolayer was formed. Then the nutrient medium was removed and 100 µL of solutions of the test drug in the given dilutions were added to the wells, which were prepared directly in the nutrient medium with the addition of 5% DMSO to improve the solubility. After 24 h of incubation of the cells with the tested compounds, the nutrient medium was removed from the plates and 100 µL of the nutrient medium without serum with MTT at a concentration of 0.5 mg/mL was added and incubated for 4 h at 37 °C. Formazan crystals were added with 100 µL of DMSO to each well. Optical density was recorded at 540 nm on an Invitrologic microplate reader (Medico-Biological Union, Novosibirsk, Russia). The experiments for all compounds were repeated three times.

The cervical cancer cell line (M-HeLa) was obtained from the Type Culture Collection of the Institute of Cytology (Russian Academy of Sciences) and the normal cell line (Chang liver) was obtained from the collection of the Research Institute of Virology of the Russian Academy of Medical Sciences (Moscow, Russia).

For flow cytometry, cells were plated 1 × 10^5^ per well and incubated for 24 h with the further addition of chitosome dispersions (2.5 mM of F2 or 1.25 mM of F3). Cellular uptake of the chitosomes was analyzed using Guava easyCyte 8HT (Luminex Corporation, Austin, TX, USA). Untreated cells were used as a negative control.

### 2.9. Animals

All experiments with animals were carried out in accordance with the Directive of the Council of the European Union 2010/63/EU. The experimental protocols were approved by the Animal Care and Use Committee of the Federal Research Center “Kazan Scientific Center of the Russian Academy of Sciences” (protocol No. 2 from 9 June 2022). Wistar rats, purchased from the Laboratory Animal Breeding Facility (Branch of Shemyakin-Ovchinnikov Institute of Bioorganic Chemistry, Puschino, Moscow Region, Russia), were kept in a well-ventilated room at 20–22 °C. The living conditions of the animals included a 12-h light/dark cycle, 60–70% relative humidity, and sufficient food and water.

### 2.10. AChE Reactivation in Rat Brain

To assess the ability of various types of chitosomes (F1, F2, F3) loaded with 2-PAM to reactivate brain AChE, a group of rats was poisoned with a sublethal dose (0.6 mg/kg) of paraoxon (POX). Brain samples for AChE activity assessment were collected 1 h after poisoning. Treatment with chitosomes with encapsulated oxime was carried out 1 h after the intraperitoneal administration of POX. 2-PAM, encapsulated in various particles, was administered intranasally to three groups of rats at a dose of 13 mg/kg. One hour after chitosome administration (i.e., 2 h after poisoning), rats were anesthetized with isoflurane inhalation and transcardially perfused with 300 mL of saline PBS (pH 7.4). Brain samples were collected, frozen in liquid nitrogen, and stored at –80 °C. Brain samples were homogenized, and AChE activity was measured according to the Ellman method [17,59]. AChE activity was expressed in relation to the total amount of protein estimated by the Bradford method [60]. There were five animals in each group of rats.

## 3. Results

### 3.1. Characterization of the Empty Chitosan-Coated Liposomes

**Figure 2:**
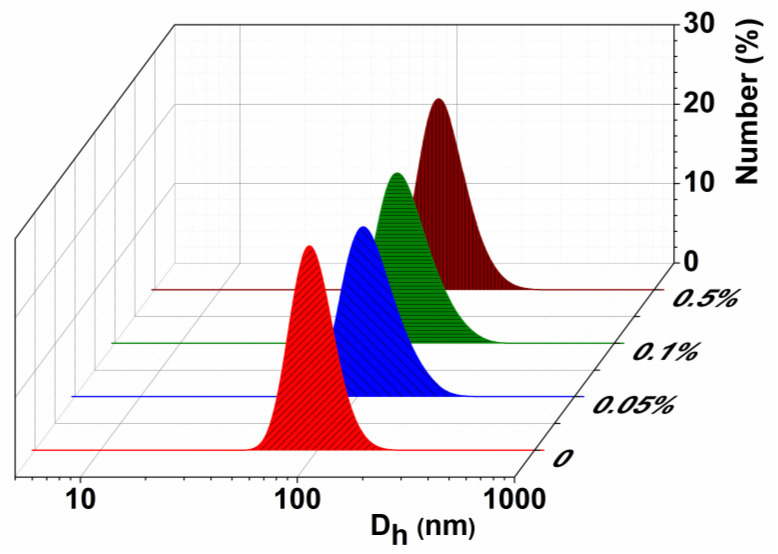
Number-averaged size distribution of the F1, at different Cs concentrations, 25 °C.

Optimization of the nanocarrier composition is one of the first and principal steps in the development of dosage forms. Therefore, the potential of several types of Cs has been studied as a polymer shell of liposomes. Chitosomes based on Cs with MW 50–190 kDa, 50 kDa, and its arginine modification are designated as F1, F2, and F3, respectively (Table 1). Physicochemical characteristics, i.e., the hydrodynamic diameter, zeta potential, and the polydispersity index of nanocarriers obtained by dynamic/electrophoretic light scattering, are presented in Table 2. The selection of the Cs concentration (sufficient to compensate and recharge the liposome surface zeta potential) is the most important step in the development of stable nanoparticles. The concentration of PC in all systems was constant (10 mM), while the polymer concentration was varied (0.05%, 0.1%, and 0.5%, i.e., 2, 8, and 16 times less than PC). The formation of monodisperse (PdI ˂ 0.2) aggregates with a hydrodynamic diameter of up to 130 nm was observed (Table 2 and Figure 2). The charge of the developed chitosomes was assessed by measuring their zeta potential. According to [31], a change in zeta potential from negative to positive is evidence of the presence of a polymer on the liposome surface. As can be seen from Table 2, the surface of liposomes was indeed recharged (from −15 mV to +20 mV), which demonstrates successful deposition of the Cs on the surface of liposomes for all selected concentrations. Modification of liposomes with cationic polymers significantly improved the stability of the formulations. Cs-decorated liposomes were stable for up to 2 months, while uncoated liposomes were destroyed after several days at room temperature.

Figure 2 shows the number-averaged size distribution of conventional liposomes and F1 chitosomes depending on the Cs concentration, which complements Table 2 and confirms the formation of hybrid lipid-Cs particles. Indeed, in the present work, for all modifications of chitosomes, an insignificant change in size is observed with an increase in the concentration of chitosan. This is likely due to the preparation method of nanocontainers, as reported in [31]. The method of extrusion through polycarbonate membranes makes it possible to obtain aggregates in a narrow size range (up to 120 nm), and the difference in the concentrations of Cs used is not large enough to see the difference in aggregate sizes. However, we noticed that the size of chitosomes significantly depends on the nature of lipid. This is consistent with the literature data [31,61] where the key role of lipid charge is reported.

**Figure 3:**
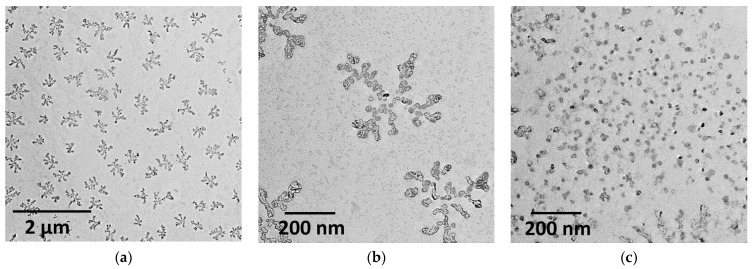
TEM- photos of empty F1 chitosomes at different scales: 2 µM (**a**); 200 nm (**b**,**c**).

Transmission electron microscopy was used to increase the evidence base in terms of particle size and shape (Figure 3). As is known, microscopic imaging requires careful sample preparation, particularly the selection of the nanoparticle concentration. Therefore, the dispersions were diluted to 5 µM relative to PC in an attempt to obtain good images. Uniformly distributed aggregates resembling dendrimers were observed throughout the field. The size of such structures is in the region of 200 nm. If each individual aggregate in this structure is evaluated, strongly aggregated small particles in the region of 20 nm are observed. At the same time, some discrepancies occurred in the results obtained by DLS and TEM, which is likely due to different concentration regimes of preparation and processing of samples.

### 3.2. Characterization of the Substrate-Loaded Chitosomes

After the stage of system optimization, the model probes RhB and AChE reactivator 2-PAM were loaded into chitosomes (containing 0.05% Cs), the physicochemical characteristics of which are presented in Table 3. The selection of the substrate concentration was chosen based on the literature data (10 and 0.5 mg/mL for 2-PAM and RhB, respectively) [29,56]. It should be noted that there were no statistical differences in the physicochemical characteristics of chitosomes in the entire concentration range of the Cs. The DLS/ELS data for chitosomes containing 0.1% and 0.5% Cs can be found in Supplementary Materials (Table S1). Both substrates have essentially no effect on the size of chitosomes. As can be seen, their D_h_ is in the nanometer range of approximately 100–130 nm with a high degree of monodispersity. During the storage of chitosomes, an increase in the zeta potential to a more positive region (up to +46 mV) was observed, while the monodispersity of the system was preserved.

**Figure 4:**
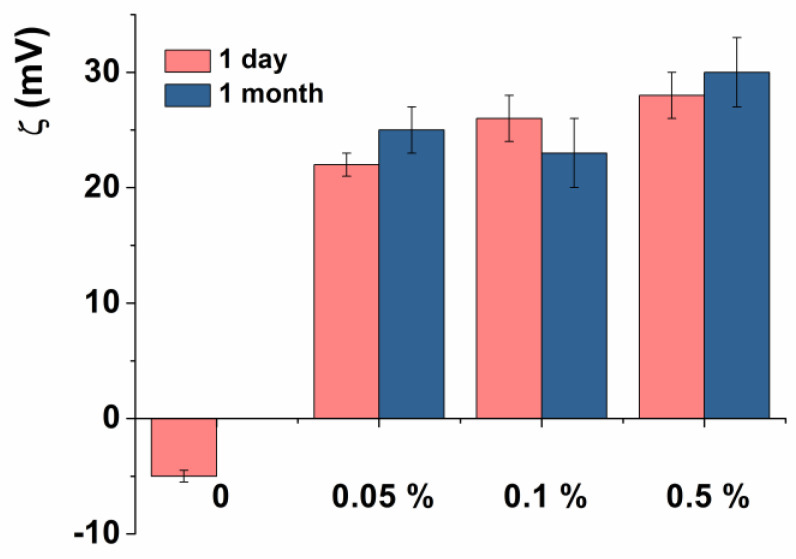
Zeta potential of RhB-loaded chitosomes, modified with various Cs concentrations during storage.

It has been demonstrated that the modification of liposomes leads to a change in the zeta potential from negative to positive values (Figure 4). A similar trend was also observed for empty chitosomes. Figure 4 and Table 3 show the dimensions and zeta potential of chitosomes during storage for 1 month at 4 °C. As can be seen, liposomes coated with Cs were significantly more stable during long-term storage than uncoated liposomes.

### 3.3. Encapsulation Efficiency (EE)

**Figure 5:**
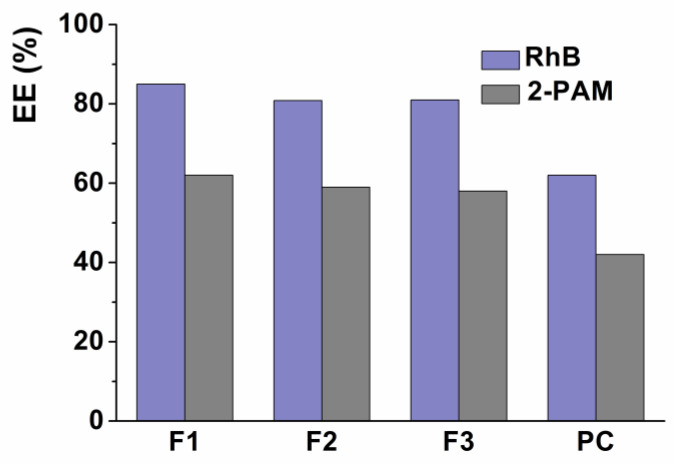
Encapsulation efficiency of RhB- and 2-PAM-loaded liposomes and chitosomes (containing 0.05% Cs).

The encapsulation efficiency of the drug is determined as the ratio of drug content within the nanocontainer to the total drug content of the suspension [31]. Since both loaded substrates (RhB and 2-PAM) are hydrophilic, the EE of chitosomes was determined by the common centrifugation method using Amicon Ultra-0.5 centrifuge filters. The encapsulation efficiency of chitosomes toward 2-PAM and RhB was approximately 60% and 85%, respectively, regardless of the molecular weight and polymer type. An increase in the Cs concentration did not lead to significant changes in the EE. It should be noted that the coating of liposomes with a polymer shell increases the EE of the aggregate by 20% compared to conventional liposomes (Figure 5).

### 3.4. In Vitro Release Study and Kinetics

**Figure 6:**
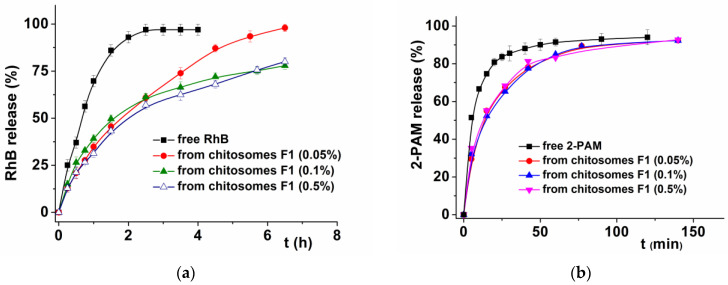
RhB (**a**) and 2-PAM (**b**) release curves in vitro from F1 chitosomes at different Cs concentrations; phosphate buffer (0.025 M), pH = 7.4, 37 °C.

It is known that in vitro study of drug release from chitosomes makes it possible to approximate their behavior in vivo. A comparative assessment of the in vitro release rate of free and encapsulated substrates was carried out by dialysis at pH 7.4, which corresponds to the physiological pH of the blood. The release curves of RhB and 2-PAM from chitosomes were studied with varying concentrations (Figure 6a,b) and modifications of the deposited Cs (Figure S2). It has been shown that the release of substrates loaded into chitosomes proceeds more slowly than free ones, which indicates the retention of the drug inside the nanovesicles. The concentration and Cs type had no significant effect on the permeability of chitosomes. Interestingly, in the case of RhB, the effect of prolonged release is more pronounced than with 2-PAM. The complete release of free RhB occurs in 2 h, while in the case of encapsulated RhB, only 50% is released during this time. In the case of 2-PAM, the release rate was faster, likely due to the greater hydrophilicity and smaller size of the substrate. During the first 5 min, 40% of 2-PAM was released instantly, which corresponds to a free substrate, just as in the case of RhB. Unlike RhB, both free and bound 2-PAM are completely released from the dialysis bag after 2 h.

The zero-order, Korsmeyer–Peppas, and Higuchi models were analyzed to determine the best kinetic model to describe the dependence of 2-PAM release from chitosomes. The correlation coefficient (R^2^), the degree of release (n), and the rate constant (k) are summarized in Table 4.

### 3.5. Analysis of Hemolytic Activity and Cytotoxicity

The hemolytic activity of nanocarriers is an important preliminary assessment of their toxicity [62]. This analysis is used to evaluate the destruction degree of red blood cells in the presence of chitosomes. The method is based on the spectrophotometric determination of the amount of hemoglobin released after erythrocyte damage. It should be noted that it is necessary to determine the toxicity of drug-loaded aggregates, since the drug may have its own hemolytic activity. It was shown that all modifications of chitosomes containing 2-PAM had low hemolytic activity in the range of 3.7–29.6%. The concentrations of chitosomes, at which 50% of erythrocytes are destroyed (HC_50_), are shown in Table 5. In addition, the cytotoxic activity of chitosomes was also determined using normal Chang liver cells and M-HeLa cervical cancer cells. The MTT test was used to study the cytotoxic effect of chitosomes. Cellular oxidoreductase enzymes are able to reduce the tetrazolium dye to insoluble blue-violet formazan, which crystallizes inside the cell. According to the results, the F2 system did not have a cytotoxic effect in the concentration range used. In the case of arginine chitosomes (F3 formulation), cytotoxicity against both cell lines increased, which indicates the lack of specificity.

### 3.6. The Cellular Uptake of RhB Loaded Chitosomes

**Figure 7:**
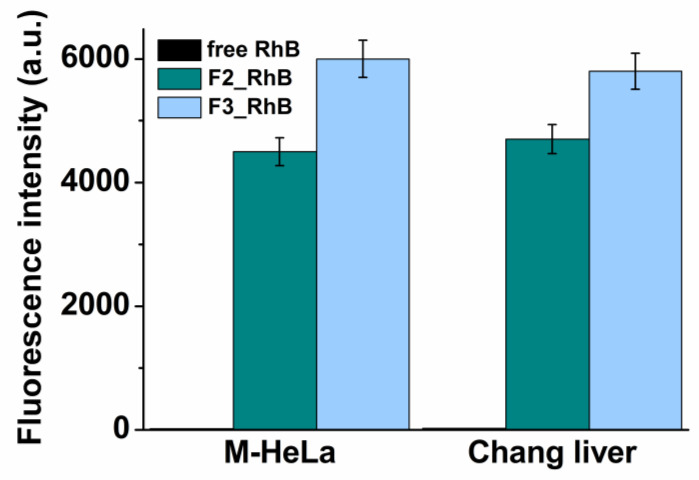
Cellular uptake study of chitosomes: (1) Free RhB; (2) F2_RhB; (3) F3_RhB after 24 h incubation.

Cellular uptake or internalization is one of the most important physicochemical criteria for nanoformulations to consider before in vivo experiments. The ability of chitosomes to penetrate the cell was determined using flow cytometry with M-HeLa and Chang liver cell lines. Normal Chang liver cells were used as models of epithelial cells. A necessary condition for determining the internalization of nanocontainers into cells is the use of fluorescent probes, in this case, RhB. Figure 7 shows the average fluorescence intensity of free RhB and encapsulated in F2 conventional and F3 arginine chitosomes. The results were evaluated 24 h after the treatment of cells with diluted solutions. According to the results, the fluorescence of the encapsulated RhB significantly exceeded the fluorescence intensity of the free dye on both cell lines. At the same time, in the case of arginine chitosomes, the fluorescence intensity was 25% higher compared to the unsubstituted Cs analogue.

**Figure 8:**
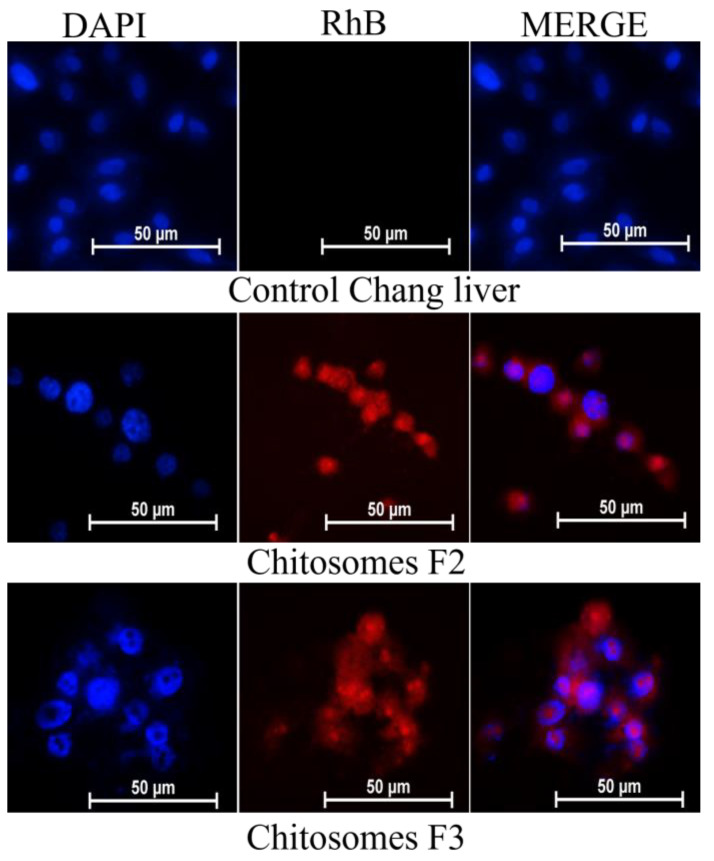
In vitro cellular uptake study of RhB-loaded chitosomes; (F2 and F3); M-HeLa cell line. Blue channel: DAPI fluorescence (nuclei); red channel: RhB fluorescence (chitosomes).

**Figure 9:**
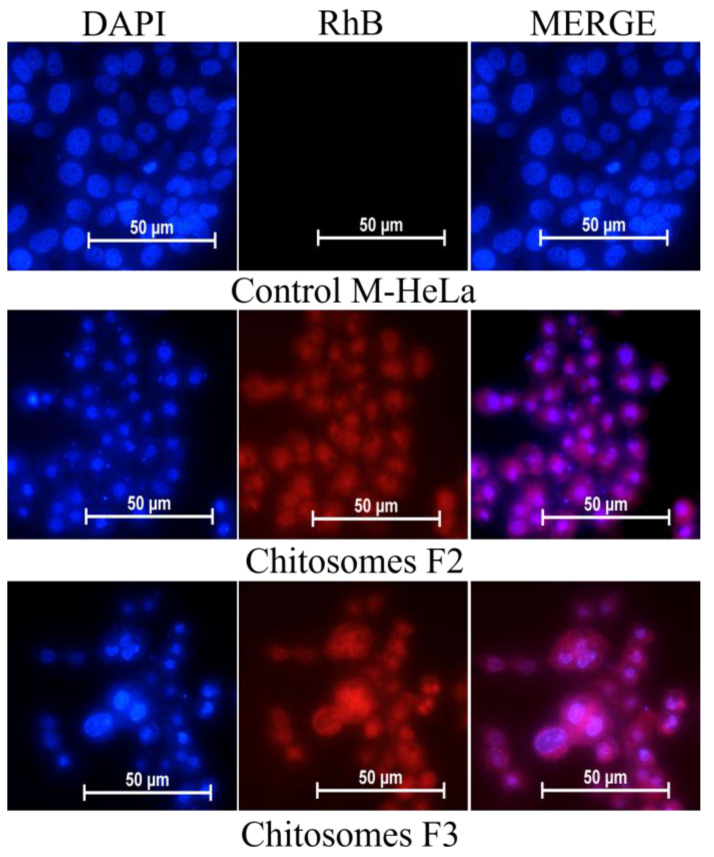
In vitro cellular uptake study of RhB-loaded chitosomes; (F2 and F3); Chang liver cell line. Blue channel: DAPI fluorescence (nuclei); red channel: RhB fluorescence (chitosomes).

The location of chitosomes within the same cell lines was studied using fluorescent microscopy. The nuclei were stained with the DNA-intercalating dye DAPI. The results obtained confirm the data obtained by flow cytometry. An intense red fluorescence of RhB was observed in the cytoplasm and nucleus of M-HeLa and Chang liver cells (Figure 8 and Figure 9).

Thus, this assay allows us to (i) test the efficacy of such kind of nanocontainers in terms of cellular uptake on model cells, (ii) compare formulations developed, and (iii) demonstrate their potentiality more generally.

### 3.7. Brain AChE Reactivation In Vivo

**Figure 10:**
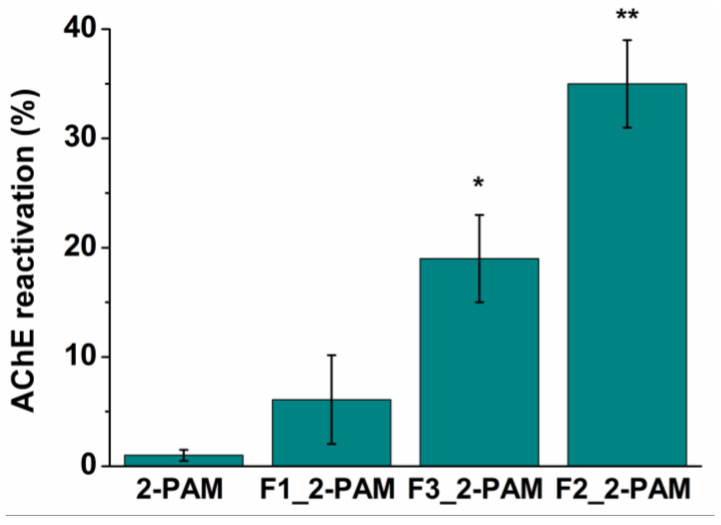
AChE reactivation rate after intranasal administration of free and formulated 2-PAM (13 mg/kg) in vivo. Data are presented as mean ± SE. (*n* = 5). * *p* = 0.017, ** *p* = 0.006 indicates significant difference by Mann–Whitney test.

The ability of all studied formulations to reactivate brain AChE was demonstrated using a paraoxon (POX) model of OP poisoning in rats in vivo. To simulate OP poisoning, rats were intraperitoneally injected with a sublethal dose (0.6 mg/kg) of the AChE inhibitor. One hour after poisoning, rats were treated with 13 mg/kg 2-PAM, and 1 h after intranasal administration of free oxime or 2-PAM-loaded chitosomes, brain samples were collected and frozen in liquid nitrogen at the same time for all treatment groups. This is explained by the fact that 2-PAM is very hydrophilic and is rapidly released from nanocontainers (more than 80% within an hour), which was shown in experiments on the release of 2-PAM in vitro (Figure 6b). In addition, in the previous work of our research group [17,29], pharmacokinetics was studied. It was shown that the maximum concentration of 2-PAM in the brain was detected after 5 min and decreased within 60 min. Therefore, the optimal time to analyze the degree of AChE reactivation in the brain of rats was 1 h after the administration of 2-PAM. The Ellman method was used for the measurement of AChE activity using UV-Vis spectrophotometry. An hour after poisoning, the activity of cerebral AChE was suppressed by 67% ± 5% without any treatment. As can be seen in Figure 10, intranasal administration of 2-PAM-loaded chitosomes (dose 13 mg/kg) reactivated brain AChE in the following order: F1 < F3 < F2 (6% < 18% < 35%), while in the case of treatment with free oxime at the same dose, AChE reactivation was 1 ± 0.7%. The values of inhibited AChE were expressed as the mean ± standard error (SE). Statistical analysis was performed using the Mann–Whitney test. *p* < 0.05 were considered statistically significant.

## 4. Discussion

The physicochemical characteristics of liposomal carriers, such as the size, zeta potential, and polydispersity index, are important indicators affecting their stability, encapsulation efficiency, cellular and tissue absorption, circulation in the bloodstream, and drug release both in vitro and in vivo. The suitability of nanoformulations for a particular route of drug administration depends on the parameters listed [63]. The freeze/thaw cycle combined with extrusion guarantees the obtainment of liposomes with sizes of approximately 120 nm with a PdI of no more than 0.1, which is extremely important for obtaining a stable dosage form [21]. It is known that with an increase in the Cs concentration, a significant increase in the size of chitosomes is often observed [35,64]. It is important that, in terms of the development of drug formulations for brain delivery, the size of the nanocarriers should be no more than 150 nm [63]. In addition, excessively high concentrations of Cs lead to a decrease in the monodispersity of liposomal dispersion. Therefore, lower concentrations of the polymer were chosen for the formation of chitosomes. Complexation between liposomes and Cs was accompanied by a slight enlargement of the particles. The dependence of the chitosome size on the MW of Cs was also shown: the higher the molecular weight of Cs, the larger the aggregates, which is consistent with the results of another research group [65]. In the case of liposomes modified with high-molecular-weight Cs (formulation F1), the hydrodynamic diameter of particles slightly increased by approximately 20 nm (Table 2). It should be noted that the complex formation between liposomes and Cs is confirmed by the monomodal particle size distribution and an increase in PdI (˂0.2; Table 2, Figure 2). In the case of other low-molecular-weight Cs (formulations F2 and F3), an increase in D_h_ and PdI was not observed. Despite the above consideration, this is also consistent with the literature data [31,61] where the key role of lipid charge is reported. The authors report that Cs has a minimal effect on the size of uncharged liposomes (egg phosphatidylcholine) compared to negatively charged vesicles, which become significantly larger with increasing Cs concentrations. This is due to the strong interaction between the polymer and the liposomal bilayer, which can lead to particle accumulation. It should be noted that in this work, we used PC with a small negative charge. The observed increase in the stability of Cs-coated liposomes may be due to the fact that the adsorbed polymer layer provides stronger repulsive interactions between the particles (steric or electrostatic) [66]. Moreover, Cs contains hydroxy-groups, which form hydrogen bonds between the polymer and water molecules. Another reason for the higher stability is related to the decrease in lipid membrane fluidity induced by Cs and the increase in the hydrophilicity of the nanoformulations.

It is known that the charge plays an important role in the intracellular uptake of nanosized vesicles, especially those bearing positive zeta potential. In addition, the zeta potential is related to the degree of repulsion between particles and, therefore, the resistance to aggregation in the system. Deposition of the polymer on the surface of liposomes led to an increase in the zeta potential of chitosomes from −15 mV to +20 mV, depending on the Cs modification (Table 2). An increase in the zeta potential of liposomes upon the Cs addition indicates that the charge is compensated, and the surface of liposomes is further recharged, which in turn demonstrates the successful formation of chitosomes. The positive charge of chitosomes is due to the protonation of the amino group present on the Cs, which forms a shell around the vesicles. An increase in the Cs concentration does not significantly affect the change in both the size and charge of chitosomes. Most likely, this is due to the saturation of the liposome surface with Cs, which is consistent with previously published works [39,40]. It should be emphasized that in the case of Cs with a molecular weight of 50–190 kDa, higher values of the zeta potential are observed compared to low-molecular-weight Cs (MW = 50 kDa). Since commercial Cs with an MW of 50–190 kDa is insoluble in water at neutral pH, the solution was prepared in an acetate buffer at pH 4.4, while the other Cs modifications were dissolved in water at pH approximately 6. The reason for such pH-dependent behavior of the polymer is the pK_a_ of the amino group of Cs, which is 6.3–6.5. In an acidic medium, the charge was created due to protonated amino groups that did not interact with the liposome surface, and an increase in pH was accompanied by a decrease in the protonated group number, which caused a significant decrease in the chitosome charge. Previously, it was shown that at pH 8.5 the charge of Cs nanoparticles is minimal [65]. The zeta potential of chitosomes was increased to a more positive region (+46 mV) one month after storage, while the monodispersity of the system was not significantly changed. It is possible that carbon dioxide may accumulate during storage, which leads to acidification of the system. In the case of Cs, acidification leads to an increase in protonation and an increase in the charge of chitosomes.

TEM images of a highly diluted chitosome solution are shown in Figure 3. The size of uniformly distributed dendrimer-like aggregates is in the region of 200 nm. If each individual aggregate in this structure is evaluated, strongly aggregated small particles in the region of 20 nm are observed. It was discussed in [67] that the aggregation of chitosomes can decrease with an increase in the Cs concentration (at least 0.8%). At higher concentrations, additional cross-links between polymers can form, which contributes to aggregation and an increase in particle size. However, dendrimer-like structures are not observed on the entire surface of the copper grid; in Figure 3c, aggregates no larger than 20 nm in size are seen over the entire field. Such aggregates were called quasi-spherical [67,68]. The phenomenon of size reduction and discrepancies in the results shown by TEM and DLS are common [40] since the sample drying, namely, the removal of water from the inner core and outer shell, can affect the reduction in particle size. In addition, the DLS also determines the hydration shell around the particles [69].

Loading the model probe RhB and the AChE reactivator 2-PAM into chitosomes did not change the D_h_ of the particles (Table 3 and Table S1). However, there are some differences in the charge characteristics since both substrates have a positive charge. Deposition of the minimum concentration of Cs (0.05%) into RhB-loaded liposomes led to an increase in the zeta potential of the system up to +34 mV (the zeta potential of empty chitosomes was +20 mV at the same concentration). However, in the case of 2-PAM-loaded chitosomes, the zeta potential remains the same as for empty ones. It is known that even small changes in ionic strength and solution pH can have a significant effect on the zeta potential [70]. The influence of NaCl and the growth of its concentration on the zeta potential of liposomes was shown [70]. Since the substrates used are salts, they were loaded into chitosomes in different amounts (see experimental data). 2-PAM has been loaded in large quantities, making it difficult to determine the physicochemical characteristics of the samples.

An important characteristic of nanocontainers is encapsulation efficiency (EE). The EE of hydrophilic molecules is regulated by the size of lipid vesicles since an increase in size leads to an increase in the volume of the liposome aqueous phase [71]. As can be seen (Figure 5), the loading of RhB and 2-PAM into chitosomes increases EE by 20% compared to conventional liposomes. This result is similar and consistent with the data of other authors [72]. The polymer likely covers the substrates that are on the liposome surface, which leads to an increase in EE [73].

One of the important tasks of the use of nanocontainers is the controlled release of the drug and the maintenance of its concentration in the blood or tissues at the desired level for as long as possible. Typically, a controlled release system first releases a portion of the contained dose in order to quickly reach an effective therapeutic drug concentration. The drug release kinetics then follow a well-defined behavior to provide a maintenance dose to achieve the desired drug concentration [74]. The dialysis method was used to evaluate the release profiles of substrates from chitosomes. Small molecules can leave the bags along the concentration gradient into the external environment, while vesicular particles remain inside due to their large size. For both substrates, the release profiles from liposomes have been studied previously [17,29]. Figure 6a,b and Figure S2 show that the dialysis process of encapsulated substrates is slower than that of free ones, which indicates their diffusion through the nanovesicle shell. In the case of RhB, there is a burst release from the dialysis bag in the first 1.5 h, followed by a slow release that lasts approximately 24 h. Burst release occurs either due to the release of non-encapsulated substrates or those present on the nanovesicle surface [75,76]. The complete release of free RhB occurs in 2 h, while in the case of encapsulated RhB, only 50% is released during this time, which indicates the delayed release. It should also be noted that higher Cs concentrations (0.1–0.5%) decrease the permeability of chitosomes. In another work, the authors showed that a high concentration of Cs slowed down the release of curcumin and caffeine and improved the stability of liposomes [72,77]. In the case of 2-PAM during the first 5 min, 40% of 2-PAM was released instantly, and it was completely released from the dialysis bag after 2 h. However, during the first hour, the release rates differ markedly. Variations in the type, molecular weight, and concentration of Cs did not lead to noticeable changes in the transmission capacity of nanocontainers. The Korsmeyer–Peppas model turned out to be the best model with the highest value of R^2^ (Table 4). The degree of release (*n*) in the Korsmeyer–Peppas equation predicts the release mechanism of the substance, i.e., *n* ˂ 0.45 corresponds to the Fickian diffusion mechanism [74,78]. The calculated values of *n* (0.33–0.38) indicate that the release of 2-PAM from chitosomes occurs by diffusion.

Hemolysis assessment is widely used in the research and development of new types of nanocontainers aimed at drug delivery [79]. It is known that Cs has a significant effect on the blood, causing the aggregation of red blood cells, leading to blood clotting [80,81,82,83]. On the one hand, this is a useful property and can be used to create topical hemostatic formulations. On the other hand, the use of Cs for administration into the bloodstream causes a number of difficulties. Intranasal administration can provide a direct route for drug formulations to the brain, but nanocontainers can enter systemic circulation. It can be seen (Table 4) that with an increase in the concentration of chitosomes with 2-PAM, an increase in hemolytic activity occurs, which is most likely associated with the presence of Cs, despite its low concentration in the stock solution (0.05%). The positively charged amino groups of Cs interact with the negatively charged plasma membrane of erythrocytes, which can provide a destructive effect [84]. The use of the arginine modification slightly reduced the hemolytic activity of chitosomes. Most likely, this is explained by the shift in the pH of the Cs solution to a more neutral region and, consequently, by the low charge of the aggregates. It was noted [79,84] that an acidic environment promotes greater hemolysis, while Cs nanoparticles dispersed in a saline solution have better compatibility with blood. In the case of evaluating the cytotoxic effect of the studied systems, arginine chitosomes showed specificity toward Chang liver and M-HeLa cells compared to unmodified chitosomes. This is perhaps a consequence of the enhanced cell penetration, which will be discussed below. The small difference in cytotoxicity between normal and cancer cells requires additional research to reduce toxicity against normal cells.

Fluorescence microscopy and flow cytometry make it possible to qualitatively and quantitatively assess the cellular uptake of nanocontainers. The developed chitosomes greatly increased the cellular uptake of RhB compared to its free form, while the efficiency of arginine chitosomes was 25% higher (Figure 7). It has previously been shown that cell-penetrating peptides are predominantly composed of arginine [49], the mechanism of cell penetration of which is still debated. First of all, there are electrostatic interactions between the guanidine group of arginine and the cell surface, which enhances cellular uptake. In addition, the guanidine group can additionally form bidentate hydrogen bonds with negatively charged phosphate, sulfate, and carboxylate groups on the cell surface [50]. Therefore, arginines are more efficient in terms of internalization than lysine, for example, but this comes at the expense of increased cytotoxicity (Table 4).

Fluorescence microscopy proves the enhanced cellular uptake of chitosomes (Figure 8 and Figure 9). The use of the DAPI dye made it possible to analyze the morphology of M-HeLa and Chang liver cells after chitosome treatment (after 24 h). The micrographs showed visible damage and a significant decrease in the size of the nuclei compared to the control. Normal cell nuclei of an oblong shape contracted and acquired a rounded shape. The addition of both systems (F2 and F3) resulted in typical signs of apoptosis, i.e., the presence of apoptotic bodies characterized by the retention of membrane isolation and bright blue fluorescence of fragmented DNA. Cell destruction by the apoptosis mechanism is favorable when nanocontainers are used, which makes it possible to use the developed nanocontainers in anticancer therapy as well [85].

The determination of AChE activity was carried out in six groups of rats: (1) control; (2) after POX poisoning (0.6 mg/kg intraperitoneally) without treatment; (3) after POX poisoning (0.6 mg/kg, intraperitoneally) with free 2-PAM treatment at a dose of 13 mg/kg (intranasally); and (4)–(6) after POX poisoning (0.6 mg/kg, intraperitoneally) with formulated 2-PAM treatment at a dose of 13 mg/kg (intranasally). As expected, upon treatment with the free form of 2 PAM at a dose of 13 mg/kg, brain AChE reactivation was low and amounted to 1% ± 0.7%. Our result is in good agreement with the previously obtained data, where a low treatment efficacy was noted with intranasal administration of free oxime at a dose of 10 mg/kg [54,86]. Additionally, Sakurada et al. have shown that with intravenous administration of pralidoxime iodide (10, 50, 100 mg/kg) without any coating, only 10% of the 2-PAM dose penetrates the brain [87]. There is no information in the literature on the reactivation of phosphylated AChE in vivo upon intranasal administration of free 2-PAM. Intranasal administration of chitosomes containing 2-PAM allowed us to reach a degree of enzyme reactivation of up to 35%, depending on the modification (Figure 10). The effectiveness of formulated 2-PAM increased in the following order: F1 < F3 < F2 (6% < 18% < 35%). For a more detailed explanation of the obtained results in vivo, additional studies are needed. Nevertheless, it can be assumed that when F1 chitosomes enter a living organism, they likely undergo changes associated with the desorption of the polymer from the liposome surface due to a change in pH. It is also worth noting that conventional liposomes showed low reactivation efficiency (7% ± 4%) even at 20 mg/kg of 2-PAM (intranasally). In the case of 2-PAM-loaded F2 and F3 formulations, Cs is water-soluble at neutral pH, so vesicles do not lose their positive charge in the nasal cavity (pH 7.4). As noted above, intranasal administration of nanovesicles can provide a direct route to the CNS through olfactory mucosa via trans- or paracellular transport [22]. According to in vitro cellular uptake results, F3 arginine chitosomes showed more efficient penetration into Chang liver cells compared to F2 chitosomes. Part of the arginine formulation likely remains inside the cells, while unmodified analogues reach the brain through tight junctions between epithelial cells. Thus, paracellular delivery is carried out to a greater extent. It was previously shown that intranasally administered cationic liposomes loaded with 2-PAM (dose 7 mg/kg) reactivated brain AChE by only 12% [54], which directly indicates the advantages of Cs-coated liposomes.

## 5. Conclusions

For the first time, mucoadhesive formulations of 2-PAM based on phosphatidylcholine, Cs of different molecular weights, and its arginine derivative were developed. The polymer shell increased the encapsulation efficiency of RhB and 2-PAM by 20% compared to conventional liposomes. The positive charge, nanometer size, and stability ensured that the particles successfully passed from the nose to the brain. Despite the identical physicochemical parameters of F2 and F3 chitosomes, the presence of arginine (F3) improved the penetration of chitosomes into normal and cancer cells, which led to an increase in cytotoxicity, but the selectivity was not significant. All chitosome modifications showed Korsmeyer–Peppas release profiles of 2-PAM via the diffusion process. The F2 composition containing 2-PAM allowed the reactivation of brain AChE by 35% in the case of paraoxon-induced poisoning in vivo. Thus, it has been shown for the first time that the application of 2-PAM-loaded chitosomes provides advanced effectiveness in intranasal oxime therapy.

## Figures and Tables

**Table 1:** DS and the elemental analysis data of Cs and ACs.

Polymer	H (%)	C (%)	N (%)	C/N (%)	DS (%)
Cs	6.70	34.53	6.86	4.96	-
ACs	5.65	36.35	5.46	6.65	28

**Table 2:** Physicochemical characteristics of Cs-coated PC liposomes, with various concentrations of polymer (0.05–0.5%) and storage time: Hydrodynamic diameter (D_h_, nm), polydispersity index (PdI), and zeta potential (ζ, mV); 25 °C.

System	C_Cs_, %	D_h_, nm	PdI	ζ, mV	D_h_, nm	PdI	ζ, mV
		1 day			2 months		
PC (10 mM)	-	106 ± 1	0.07 ± 0.01	−15 ± 1	Not stable		
F1	0.05	131 ± 1	0.17 ± 0.01	18 ± 1	134 ± 1	0.17 ± 0.01	37 ± 1
F1	0.1	135 ± 1	0.19 ± 0.01	17 ± 1	131 ± 1	0.14 ± 0.01	35 ± 1
F1	0.5	128 ± 1	0.16 ± 0.01	20 ± 1	135 ± 1	0.28 ± 0.02	46 ± 1
F2	0.05	110 ± 1	0.05 ± 0.01	5 ± 1	109 ± 1	0.07 ± 0.02	8 ± 1
F2	0.1	111 ± 2	0.06 ± 0.04	5 ± 1	111 ± 1	0.07 ± 0.02	11 ± 1
F3	0.05	111 ± 2	0.06 ± 0.01	4 ± 1	111 ± 1	0.07 ± 0.01	22 ± 1
F3	0.1	115 ± 1	0.08 ± 0.01	5 ± 1	115 ± 1	0.08 ± 0.01	19 ± 1

**Table 3:** Physicochemical characteristics of RhB- or 2-PAM-loaded liposomes and chitosomes, modified with various polymers (0.05%) and storage time: hydrodynamic diameter (D_h_, nm); polydispersity index (PdI), and zeta potential (ζ, mV).

System	C_Cs_, %	D_h_, nm	PdI	ζ, mV	D_h_, nm	PdI	ζ, mV
RhB							
		1 day			1 month		
PC (10 mM)	-	105 ± 2	0.10 ± 0.02	−5 ± 1	Not stable		
F1	0.05	131 ± 1	0.10 ± 0.03	22 ± 1	133 ± 1	0.12 ± 0.01	29 ± 1
F2	0.05	119 ± 1	0.19 ± 0.01	32 ± 1	121 ± 1	0.21 ± 0.01	25 ± 1
F3	0.05	100 ± 1	0.12 ± 0.01	34 ± 1	115 ± 1	0.13 ± 0.09	30 ± 1
2-PAM							
PC (10 mM)	-	118 ± 1	0.06 ± 0.01	−7 ± 1	Not stable		
F1	0.05	134 ± 1	0.06 ± 0.01	9 ± 1	134 ± 1	0.13 ± 0.01	16 ± 1
F2	0.05	113 ± 1	0.07 ± 0.01	6 ± 1	116 ± 1	0.07 ± 0.02	11 ± 1
F3	0.05	116 ± 1	0.07 ± 0.01	6 ± 1	115 ± 1	0.08 ± 0.01	7 ± 1

**Table 4:** Kinetic models of 2-PAM release from chitosomes in vitro.

Kinetic Model	Parameters	Chitosomes		
		0.05%	0.1%	0.5%
Zero order $F=k_{0}\cdot t$	k_0_	1.755	1.745	1.774
	R^2^	0.588	0.606	0.501
Higuchi Model $F=k_{n}\cdot t^{1/2}$	k_n_	12.008	11.916	12.209
	R^2^	0.964	0.973	0.943
Korsmeyer–Peppas Model $F=k_{KP}\cdot t^{n}$	k_KP_	18.737	18.535	22.033
	n	0.376	0.377	0.336
	R^2^	0.987	0.997	0.989

where R^2^—correlation coefficient; F is the fraction of the substrate released at time t; k_0_ is the zero-order release constant; k_n_ is the Higuchi release constant; k_KP_—release constant, taking into account the structural and geometric characteristics of the dosage form; n is the degree of diffusion release.

**Table 5:** Hemolytic activity and cytotoxicity of 2-PAM-containing chitosomes: The concentration of Cs is 0.05%.

System	C_PC_, mM	Hemolysis, %	HC_50,_ mM *	IC_50_, mM * Chang Liver/M-HeLa
F1_2-PAM	5	29.6	>5	
	2.5	20.7		
	1.25	7.1		
	0.625	4.6		
F2_2-PAM	5	23.6	>5	>5/>5
	2.5	16.6		
	1.25	7.8		
	0.625	4.5		
F3_2-PAM	5	22.3	>5	3.5/3
	2.5	16.6		
	1.25	8.2		
	0.625	3.7		

* PC concentration.

## Data Availability

Not applicable.

## Supplementary Materials

The following supporting information can be downloaded at: https://www.mdpi.com/article/10.3390/pharmaceutics14122846/s1, Figure S1: FTIR spectra of arginine modified chitosan, plain chitosan and arginine; Figure S2: 2-PAM release curves in vitro from chitosomes, modified with various polymers; phosphate buffer (0.025 M), pH = 7.4, 37 °C; Table S1: Physicochemical characteristics of liposomes and chitosomes with 2-PAM, at various concentration and type of Cs: encapsulation efficiency (EE, %), hydrodynamic diameter (D_h_, nm); polydispersity index (PdI), zeta-potential of particles (ζ, mV).
