# Supplementary material for: Therapy of Organophosphate Poisoning via Intranasal Administration of 2-PAM-Loaded Chitosomes
Source: Pharmaceutics. 2022 Dec 19;14(12):2846. doi: 10.3390/pharmaceutics14122846 (PMC9781263; doi:10.3390/pharmaceutics14122846)
Supplement: Supplementary file 1 [file pharmaceutics-14-02846-s001.zip › pharmaceutics-2029033-supplementary.pdf]

pharmaceutics

MDPI

Supplementary Materials

Therapy of organophosphate poisoning via intranasal administration of 2-PAM-loaded chitosomes

Elmira A. Vasilieva *, Darya A. Kuznetsova, Farida G. Valeeva, Denis M. Kuznetsov, Andrey V. Zakharov, Syumbelya K. Amerhanova, Alexandra D. Voloshina, Irina V. Zueva, Konstantin A. Petrov, Lucia Ya. Zakharova

Arbuzov Institute of Organic and Physical Chemistry, FRC Kazan Scientific Center, Russian Academy of Sciences, Arbuzov Str. 8, 420088 Kazan, Russia

* Correspondence: vasilevaelmira@mail.ru.

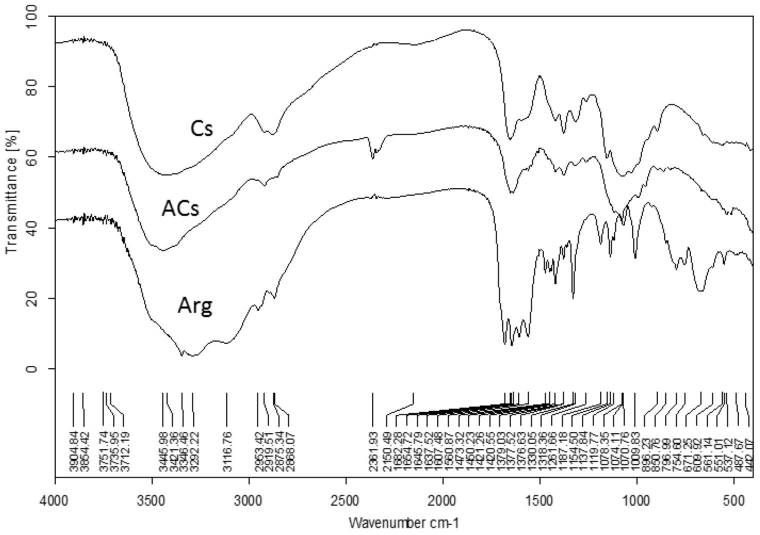

Figure S1. FTIR spectra of arginine modified chitosan, plain chitosan and arginine.

Table S1. Physicochemical characteristics of liposomes and chitosomes with 2-PAM, at various concentration and type of Cs: encapsulation efficiency (EE, %), hydrodynamic diameter (D_h, nm); polydispersity index (PdI), zeta-potential of particles (ζ, mV).

System	C_{Cs}, %	EE (%)	D_h, nm	PdI	ζ, mV	D_h, nm	PdI	ζ, mV
		1 day				1 month		
PC (10 mM)	-	40±1	104±1	0.06 ± 0.01	-7.4±0.1	Not stable		
F1	0.1	67±1	134±1	0.06±0.21	9±1	134±1	0.13±0.01	16±1
F1	0.5	65±1	155±1	0.11±0.02	12±1	143±2	0.25±0.02	37±1
F2	0.1	59±3	113±1	0.07±0.01	6.2±0.1	116±1	0.07±0.02	11±1
F2	0.5	63±1	110±1	0.05±0.01	5.1±0.6	110±1	0.07±0.01	16±1
F3	0.1	58±3	116±3	0.07±0.01	3.5±0.1	115±1	0.09±0.01	19±1
F3	0.5	62±1	114±1	0.06±0.02	4.3±1.6	113±2	0.12±0.01	23±1

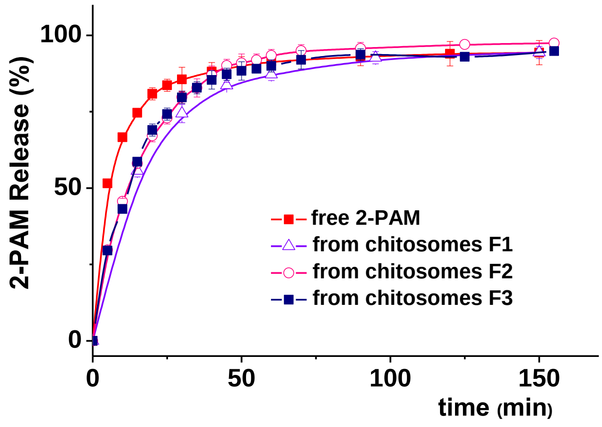

Figure S2. 2-PAM release curves in vitro from chitosomes, modified with various polymers; phosphate buffer (0.025 M), pH = 7.4, 37 °C.
